# Examining diffusion to understand the *how* of SASA!, a violence against women and HIV prevention intervention in Uganda

**DOI:** 10.1186/s12889-018-5508-4

**Published:** 2018-05-11

**Authors:** Elizabeth Starmann, Lori Heise, Nambusi Kyegombe, Karen Devries, Tanya Abramsky, Lori Michau, Tina Musuya, Charlotte Watts, Martine Collumbien

**Affiliations:** 1 Department of Global Health and Development, London School of Hygiene & Tropical Medicine, Keppel Street, London, WC1E 7HT UK; 2 Department of Population, Family and Reproductive Health, Johns Hopkins Bloomberg School of Public Health, 615 N Wolfe Street, Baltimore, MD 21205 USA; 3 Raising Voices, Plot 16 Tufnell Drive, Kampala, Uganda; 4 Center for Domestic Violence Prevention, Plot 16 Tufnell Drive, Kampala, Uganda; 5 Department of Social & Environmental Health Research, London School of Hygiene & Tropical Medicine, Keppel Street, London, WC1E 7HT UK

**Keywords:** Sub-Saharan Africa, Behaviour change, Diffusion, Social networks, Community mobilisation, Violence against women, Partner violence, SASA!, Uganda, Change agent

## Abstract

**Background:**

A growing number of complex public health interventions combine mass media with community-based “change agents” and/or mobilisation efforts acting at multiple levels. While impact evaluations are important, there is a paucity of research into the more nuanced roles intervention and social network factors may play in achieving intervention outcomes, making it difficult to understand how different aspects of the intervention worked (or did not). This study applied aspects of diffusion of innovations theory to explore how SASA!, a community mobilisation approach for preventing HIV and violence against women, diffused within intervention communities and the factors that influenced the uptake of new ideas and behaviours around intimate partner relationships and violence.

**Methods:**

This paper is based on a qualitative study of couples living in SASA communities and secondary analysis of endline quantitative data collected as part of a cluster randomised control trial designed to evaluate the impact of the SASA! intervention. The primary trial was conducted in eight communities in Kampala, Uganda between 2007 and 2012. The secondary analysis of follow up survey data used multivariate logistic regression to examine associations between intervention exposure and interpersonal communication, and relationship change (*n* = 928). The qualitative study used in-depth interviews (*n* = 20) and framework analysis methods to explore the intervention attributes that facilitated engagement with the intervention and uptake of new ideas and behaviours in intimate relationships.

**Results:**

We found communication materials and mid media channels generated awareness and knowledge, while the concurrent influence from interpersonal communication with community-based change agents and social network members more frequently facilitated changes in behaviour. The results indicate combining community mobilisation components, programme content that reflects peoples’ lives and direct support through local change agents can facilitate diffusion and powerful collective change processes in communities.

**Conclusions:**

This study makes clear the value of applying diffusion of innovations theory to illuminate *how* complex public health intervention evaluations effect change. It also contributes to our knowledge of partner violence prevention in a low-income, urban East African context.

**Trial registration:**

ClinicalTrials.gov #NCT00790959. Registered 13th November 2008.

## Background

Violence against women (VAW) is a major human rights and public health concern, with significant impacts on women’s health [1, 2], including increased vulnerability to HIV [3, 4]. Intimate partner violence (IPV) is the most common form of violence against women, with 30% of women globally experiencing it during their lifetime [1]. There is now a growing body of evidence on the impact of prevention interventions on HIV and IPV in different contexts. In South Africa, the IMAGE Study assessed the impact of a combined microfinance programme and participatory gender and HIV training [5]. The cluster randomized study found that the intervention led to a 55% reduction in past year IPV over two years and among younger participants was associated with increases in HIV testing and reduced prevalence of unprotected sex at last intercourse with a non-spousal partner [6]. The programme has been scaled up in South Africa and is currently being replicated in Tanzania as part of the MAISHA study [7]. Stepping Stones, a participatory HIV prevention programme which includes gender, relationship education and IPV content, has been used throughout Africa and evaluated extensively. A cluster randomized control study in South Africa found that after a two year follow up period there was no association between the intervention and a reduction in HIV, but there was an association with a 33% reduction in HSV-2 incidence. Reductions were also observed among men in reported levels of IPV perpetration against women and sexual risk behaviours [8]. And, more recently a randomized control trial of the Bandebereho gender-transformative couples’ intervention in Rwanda found that compared to the control group, women in the intervention group reported less past-year physical (OR 0.37, *p* < 0.001) and sexual IPV (OR 0.34, *p* < 0.001) [9]. The program engages men and their female partners (for some sessions) on topics including gender and power, IPV, couple communication and parenting.

There has also been wide recognition that a range of public health issues centred within intimate relationships, such as HIV and partner violence, are influenced by broader community and societal factors [10–12]. In response a growing number of complex interventions combine mass media with community mobilisation efforts and/or community-based change agents to intervene at multiple levels [11, 13, 14]. Programme H a participatory intervention with young men uses interactive group education sessions and community wide social marketing to address the acceptability of violence among young men and transitional norms of masculinity. A quasi-experimental study in Brazil found significant reduction in reported inequitable gender norms, a decrease in reported STI symptoms, and an increase in reported condom use at last sex with a primary partner was observed after one year [15]. SASA!, a combined HIV and VAW prevention programme uses multiple channels to catalyse community-led change of norms and behaviours that perpetuate gender inequality, violence and increased HIV vulnerability for women [16]. It does this through engaging health workers and local authorities and training community activists (CA) who introduce concepts during informal activities in their communities using communication materials, media and advocacy and community based support over time. Others, such as Soul City and Sexto Sentido, use ‘edutainment’ to reinforce social change messages around HIV and partner violence through mass media messaging and radio and television dramas [13]. Rigorous trials in Sub-Saharan Africa have also suggested community mobilisation and reflective strategies work to prevent IPV [17, 18].

Research evaluating such interventions tends to focus on measuring their impact on intended outcomes and, in some cases, on establishing whether exposure to the intervention follows a typical dose response curve. While this is important, there is a paucity of research into the more nuanced roles intervention and social network factors may play in achieving these outcomes, making it difficult to understand how different aspects of the intervention worked (or did not), and how it could be improved or best adapted in different contexts. For example, while a number of studies on the Stepping Stones HIV intervention found it to be effective, Bradley et al.’s [19] study on the diffusion of the intervention among social networks revealed important weaknesses. They observed that while Stepping Stones aims to have a community level effect, there was only diffusion of knowledge among personal contacts and limited community level diffusion. Examining diffusion provided key insights into how the intervention could further strengthen diffusion, increasing impact.

Diffusion of innovations theory provides a useful framework for exploring how attributes of the individual, intervention, and social system, converge to allow the spread of new ideas/behaviours from a source (e.g. implementing organisation) to an individual (e.g. community members) via different communication channels (e.g. mass media, interpersonal communication) and influence [20]. In this paper we use data from a multidisciplinary evaluation of SASA! to gain understanding of the communication channels and intervention attributes through which IPV interventions can stimulate behaviour changes in intimate relationships. The SASA! Study was conducted in Kampala, Uganda between 2008 and 2012 and comprised a cross-sectional cluster randomised control trial (RCT) [21], qualitative studies [22], a process evaluation and a costing study [23]. The RCT showed the intervention to be associated with lower acceptability of IPV, as well as reductions in women’s experiences of IPV—past year experience of all types of IPV was lower in intervention compared to control communities, with statistically significant effects observed for past year experience of high intensity emotional aggression and controlling behaviours, and cessation of physical, sexual and emotional IPV where it was previously occurring [24]. The aim of this paper is to examine through which communication channels SASA! diffused and the intervention attributes that contributed to its effect. Specifically, we analyse the associations between different communication channel exposures and reported positive change in relationship quality (as it is on the pathway to IPV cessation) from our larger quantitative survey sample. Qualitative data is examined to elucidate the intervention attributes that facilitated engagement with the intervention and uptake of new ideas and behaviours in intimate relationships.

### Diffusion of innovations theory

Diffusion of innovations theory focuses on the role different communication channels play in facilitating individuals’ ‘exposure’ (both ‘direct’ and ‘indirect’) to new ideas and their movement through a ‘innovation-decision process’ (knowledge, persuasion, decision, implementation, confirmation) [20]. The term ‘diffusion’ in this context (and applied in this paper) includes not only the spreading of new ideas, but the entire process from direct or indirect exposure to adoption. ‘Adoption’ is defined as the uptake of the innovation, ideas or programme by the targeted audience [20]. The theory has been applied in a variety of ways across public health—in particular in HIV prevention [13] and family planning [25, 26]—and there is empirical support for aspects of the theory in the broader public health literature [20, 27]. It has not—to our knowledge—been applied to partner violence prevention interventions.

SASA! is designed to diffuse new ideas and behaviours to community members directly through, 1) mass media channels: TV, radio and posters displayed in shops, on gates, at local authority offices, health centres and in the market; 2) mid media channels: videos or dramas performed in public spaces in the community; and, 3) interpersonal communication with change agents: quick chats, community conversations and card games facilitated by community activists trained in SASA!. SASA! also anticipates that community members will be indirectly exposed to messages through ‘interpersonal communication’ among social network members about SASA! (e.g. peers, neighbours, elders). According to the theory mass and mid media channels are most effective in generating awareness, identification and knowledge about new ideas and behaviours [20, 26]. Interpersonal communication about the new ideas is, in turn, influential in persuading individuals to adopt or reject new behaviours [25, 28] and those more ‘homophilious’ or similar, such as peers, have the greatest sway [20].

Diffusion theorists have also identified key attributes of interventions or innovations that influence how quickly new ideas or behaviours are adopted [29] and account for most of the variation between innovations that are adopted quickly and those that are not [20]. For example, individuals need a sense that there is a ‘relative advantage’ to the new ideas or behaviours: a perceived personal, physical, social or economic benefit. Next, research has found people often carry out a small trial first to test out the relative advantages of a new behaviour or smaller change towards it before deciding to adopt (‘trialability’). It also needs to be compatible with their life, their perceived or ‘felt needs’ and existing sociocultural values (‘compatibility’). And, the perceived ‘complexity’ of applying new ideas and behaviours can influence how willing individuals are to try them. New behaviours are also more likely to be diffused if they are easily observed by others (‘observability’). Witnessing the positive experience and changes in others encourages individuals to try new behaviours/innovations themselves.

Change agents are also evidenced to play an influential role in adoption. Rogers outlines seven roles a change agent ideally plays in introducing new ideas and behaviours within communities and facilitating adoption: develop a need for change; establish an information exchange relationship; diagnose problems; create an intent to change in individuals; translate intent into action; stabilise adoption and prevent discontinuation; and, achieve a terminal relationship by developing community members’ capacity to be their own change agents [20].

Together the communication channels, intervention attributes and change agent roles can serve as a guide or starting point when evaluating interventions, and help illuminate what facilitated or prevented the intervention’s intended outcomes. In this paper we investigate three research questions using quantitative and qualitative data: 1) Through what communication channels is SASA! diffusing in intervention communities? 2) Is there a relationship between an individual’s specific communication channel exposure (e.g. mid media, interpersonal communication with peers or change agents) and experiencing positive changes in their relationship since being exposed to SASA!? 3) What intervention attributes facilitated engagement with SASA! and uptake of new ideas and behaviours in intimate relationships?

## Methods

### Study site and SASA! Intervention

The SASA! approach was developed by Raising Voices and implemented by the Center for Domestic Violence Prevention (CEDOVIP). The SASA! study was conducted in eight high-density, impoverished communities in Kampala, Uganda. Rates of HIV and IPV are high in Kampala, with 9.5% of women and 4.1% of men aged 15-49 estimated to be living with HIV and 45% of ever-married women reporting IPV at some point in their lives [30]. Partner violence is closely linked to the changing gender roles and expectations around relationships in Uganda, as well as alcohol use and multiple sexual partners [31, 32].

SASA! is a community mobilisation approach for preventing VAW and HIV. It is designed for catalysing community-led change of norms and behaviours that perpetuate gender inequality, violence and increased HIV vulnerability for women. SASA! means ‘Now’ in Kiswahili and is an acronym for the four phases of the approach - Start, Awareness, Support, Action. In the Start phase, an organization using *SASA!* begins by orienting staff to the approach and key concepts of power. They then select an equal number of female and male community activists (CAs)—well-known and respected people in the community (i.e. ‘opinion leaders’) selected for their interest in issues of violence, power and rights—and similarly select institutional activists, for example, from police, health care, local government and faith-based groups. All activists are introduced to the new ways of thinking about power and power imbalances in their own lives and within the community, and are mentored in the *SASA!* approach.

With the support of staff, the activists then take the lead as the approach moves forward into the Awareness, Support and Action phases. In these phases, the activists lead informal, benefits-based activities within their existing social networks—fostering open discussions, critical thinking and supportive person-to-person and public activism among their families, friends, colleagues and neighbours. Together, they introduce the community and its institutions to the new concepts of power, encouraging a gendered analysis of power imbalances through four strategies: Local Activism, Media and Advocacy, Communication Materials, and Training. The combination of these strategies ensures that community members are repeatedly exposed to *SASA!* ideas in diverse ways within the course of their daily lives, from people they know and trust as well as from more formal sources within the community. Each phase builds on the other and addresses a different concept of power, with an increasing number of individuals and groups involved, strengthening a critical mass committed and able to create social norm change [33].

### Study design

**Fig. 1:**
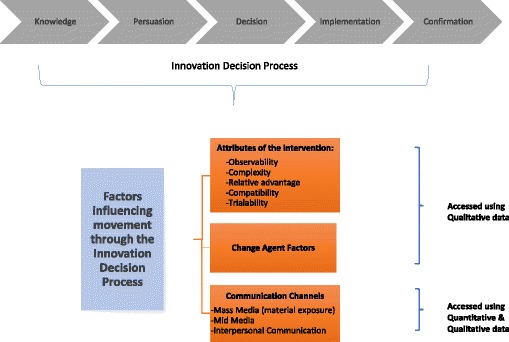
Constructs from Diffusion of Innovation theory measured in the quantitative and qualitative data analyses

This paper used both qualitative and quantitative data to extend the breadth and depth of understanding of diffusion within the context of the SASA! intervention. The quantitative and qualitative analyses were integrated to achieve complementarity through answering related questions using the type of data most suited to each question. Figure 1, presents a diagram of diffusion of innovations theory and indicates which constructs were analysed with each data set for this paper. Aspects of the theory such as movement through the innovation decision process could not be examined due to limitations of cross sectional data. The length of the survey instrument did not allow us to measure all constructs quantitatively, and some were explored in the qualitative component exclusively.

### Quantitative

**Fig. 2:**
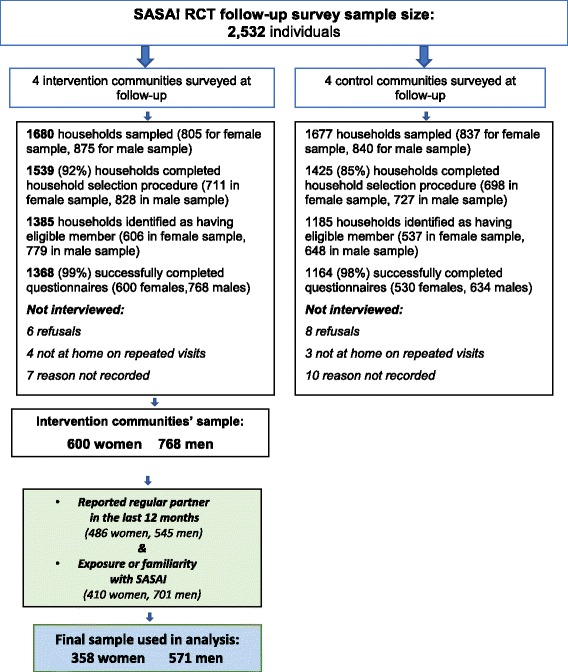
Quantitative sampling diagram

The data for this analysis was collected during the follow-up survey of the SASA! RCT, described elsewhere in detail [34]. Briefly, the survey was conducted in 2012 with 2532 community members in eight sites (four intervention, four control) following 2.8 years of programming. A person was eligible for inclusion in the survey if they usually lived in the household and shared food, had lived in the area for at least a year, and were 18-to 49-years old. A limit of one respondent per household was set out to protect respondent safety and confidentiality. The sample for this analysis was restricted to reflect the focus on relationship change linked to intervention exposure. Thus, it only included participants living in intervention communities who reported having a regular partner in the last twelve months and having had exposure or familiarity with SASA! (*n* = 929, with 358 women and 571 men) (Fig. 2). In the full dataset, 81% of men and 84% of women had a regular partner, and 91% of men and 68% of women in intervention communities reported SASA! exposure.

#### Outcomes and exposure variables

This analysis explored how exposure to SASA! through different communication channels (e.g. mid media, interpersonal communication) were associated with reporting positive change in the relationship since exposure to SASA!. This outcome was chosen as a proxy for movement on the continuum of change towards improved relationship quality and less violence based on the hypothesis that positive change in intimate partner relationships leads to reductions in IPV [35]. It was measured by asking: 1. “Has anything changed in your relationship with your partner since you became involved in SASA!?” If they answered yes, they were asked, 2. “Did the changes include a. better communication, b. increased discussion on important decisions in the household, c. more closeness, d. more respect?” Nearly all respondents that reported yes to question one answered yes to each item in question two. Thus, question one was used in this analysis as the indicator of positive change in the relationship resulting from SASA! exposure.

The exposure variables were chosen as indicators of the communication channels through which SASA! messaging may diffuse either directly from intervention exposure or indirectly via discussion about SASA! with different social network members. During the RCT the main forms of mass media in the SASA! Activist Kit (radio/TV) were not used to prevent contamination to control communities. Thus, for this analysis we measured material exposure (posters, comics, picture cards, information sheets), mid media exposure (dramas, audio plays), interpersonal communication (discussion activities with change agent, seeking change agent support, discussion about SASA! with social network members) and multi-channel exposure (having material *and* mid media/activity exposure) (Table 1). The analysis examined the independent effects of each type of intervention exposure separately as well as the effect of communication about SASA! among different social network members. It also tests the hypothesis that exposure to multiple channels (materials plus drama and/or discussion activities) would yield stronger associations with the outcome of interest than only materials exposure.

**Table 1:** Exposure variables and associated follow-up survey items

Exposure Variables	Survey item^a^	Categories of exposure^a^:
Intervention exposure		
*Communication materials*	“*How many times have you seen any of these materials about violence against women and relationships between men and women?”* (the interviewer showed them a card with illustrations of SASA! posters, comics, picture cards, information sheets).	Categorical variable: - 0-1 *(reference group)* −2-5 - > 5
*Mid media*	*How many times have you been to a SASA!/CEDOVIP film, drama or listened to an audio play in your community about violence against women and relationships between women and men?*	Categorical variable: -never *(reference group)* -once -a few 2-5 -many > 5
*Interpersonal communication* -at discussion activity w/ change agent	*How many times have you been to an activity or quick chat in your community where you looked at one of the SASA!/CEDOVIP materials (poster, comic, or picture card, etc) and talked about violence against women and relationships between women and men?*	
-*Sought CA advice*	*How many times have you sought advice from a SASA! community activist?*	Binary variable -never *(reference group)* −1 or more times
Interpersonal Communication with different social network members:		
-*Talked with Elders*	*I) Have you talked with your* *parent* *about SASA!? If yes: II) How many times?*	Categorical variable^b^: -low (0-2 times) *(reference group)* -medium (3-5 times) -high (> 5 times)
	*I) Have you talked with your* *in-law* *about SASA!? If yes: II) How many times?*	
	*I) Have you talked with an* *elder* *about SASA!? If yes: II) How many times?*	
*-Talked with Peers*	*I) Have you talked with a* *friend* *about SASA!? If yes: II) How many times?*	
	*I) Have you talked with a* *neighbour* *about SASA!? If yes: II) How many times?*	
*-Talked with Partner*	*I) Have you talked with your* *partner* *about SASA!? If yes: II) How many times?*	
*Multi-channel exposure*	“*How many times have you seen any of these materials about violence against women and relationships between men and women?”* (the interviewer showed them a card with illustrations of SASA! materials).	Categorical variable: -materials (mass media) exposure only *(reference group)* -low ‘multi-channel’ exposure (exposed 1-4 times to activities and/or dramas/films) -high ‘multi-channel’ exposure (exposed > 5 times to activities and/or dramas/films)
	*How many times have you been to a SASA!/CEDOVIP film, drama or listened to an audio play in your community about violence against women and relationships between women and men?*	
	*How many times have you been to an activity or quick chat in your community where you looked at one of the SASA!/CEDOVIP materials (poster, comic, or picture card, etc) and talked about violence against women and relationships between women and men?*	

^a^To measure dose-response relationships frequency of exposure was captured using 4 categories: never, once, a few times (2-3), or many times (5+). Some variables were re-coded or re-categorised for statistical reasons for the regression analysis

^b^Composite frequencies based on frequencies with which they spoke to each type of person

#### Statistical analysis

The statistical analysis was conducted using STATA version 13.1. All analyses were conducted separately for men and women given that gendered variation in response patterns are frequently observed in IPV research [18]. Clustering of the outcomes within the study sites was ‘small’ (< 0.1 intraclass correlation), hence the analysis did not adjust for the clustered sampling design [36]. For each outcome, logistic regression was used to calculate unadjusted odds ratios (and 95% confidence intervals (CI)) comparing odds of the outcome in each of the higher exposure categories with that in the lowest exposure category. The likelihood ratio test (LRT) was used to compare models with and without each exposure.

Multivariate logistic regression was used to further explore associations between the exposures and outcome. We first modelled the association between multi-channel exposure and the outcome, adjusted for potential confounders (age, marital status, socioeconomic status (SES) and education level). Separate models were then used to explore the independent effect of each of the single-channel exposures on the outcome, adjusted for the other channels of exposure and potential confounders. Variables for inclusion in the model were decided upon a priori based on conceptual considerations, however models were also checked for collinearity problems. As with the unadjusted analysis, 95% confidence intervals were calculated to estimate the precision of the adjusted odds ratio (aOR), and the overall *p*-value for each exposure generated using the LRT to test for model fit.

### Qualitative

Using data from the SASA! couples study, detailed elsewhere [37], the qualitative analysis examined participants’ engagement with the intervention and how different communication channel exposures to SASA! influenced relationship changes. Participants in the couples’ study were sampled purposively from the RCT follow up survey respondents. RCT participants that agreed to be contacted again were sampled using the following criteria: in current relationship since 2010 or before; IPV reported before the last 12 months, but not in the last 12 months; exposure to SASA! of any intensity (note their partner may not have been exposed); and, positive change in relationship since becoming involved in SASA!. Those reporting violence in the last twelve months were not selected as a safety precaution as interviewing them could incite further violence (i.e. if the man thought his partner had “told” on him). Initial efforts to recruit couples through contacting female RCT participants yielded only two couples. Therefore, eight couples were recruited through male RCT participants with further precautions taken to ensure their female partners were not pressured into participating. Couples were sampled between August-October 2012 from across the four intervention communities, with each partner interviewed separately using a semi-structured interview tool (20 interviews in total; 10 women and 10 men). The guide starts with general questions about the participant’s relationship and any changes they have observed. This allowed participants to first mention SASA! of their own accord as well as attribute any changes in their relationship to it (or not). Later in the guide there are more specific questions and probes about SASA! exposure and how it impacted their relationship. A participatory timeline was used to help with recall. Interviews were conducted, transcribed and translated from Luganda to English by bi-lingual research assistants and data were entered into NVIVO 10 software for coding and analysis by the first author. While couples were sampled, the unit of analysis for this investigation is the individual. Data were analysed using framework analysis, a method that allows the researcher to systematically organize and compare ‘raw’ data by theme and case using a framework matrix linked to the original transcripts [38].

The World Health Organisation protocol for interviewing women on IPV was observed [39] and each participant gave individual written informed consent to be interviewed and, in the qualitative study, to be audio recorded. The SASA! Study received ethical clearance from Institutional and National Review Boards. Couples were numbered with partners indicated by M for male, F for female (e.g. 1F, 1 M) and pseudonyms used to protect confidentiality.

## Results

Detailed characteristics of the quantitative sample including intervention exposure are presented in Table 2. The majority of men and women lived in rented homes with access to electricity; water was from a public tap and sanitation facilities were mainly pit latrine toilets. The mean age was 28 for women and 29 for men. The largest proportion (35%) were Catholic, followed by Muslim and Protestant (25% each). The majority were literate (96% men, 89% women) and educated above the primary level (71% men, 66% women). 32% of men completed secondary school or higher compared with 20% of women. 93% of men versus 61% of women were employed. 83% of women and 65% of men had children and 39 and 17% respectively had three or more.

**Table 2:** Characteristics of the sample (i.e. partnered people with any SASA! exposure)

	Male (*N* = 571)		Female(*N* = 358)	
Household & individual level:	n (%)		n (%)	
Electricity in home	506	(89%)	297	(83%)
Water source: outside/public tap	457	(85%)	291	(81%)
Toilet facility: ventilated/traditional pit latrine	530	(93%)	299	(84%)
Lives in rented housing	461	(81%)	268	(75%)
Age group	*mean = 29*		*mean = 28*	
18-24 yrs	161	(28%)	128	(36%)
25-34 yrs	258	(45%)	171	(48%)
35-49 yrs	152	(27%)	59	(17%)
Lived in community more than 3 years	462	(81%)	221	(62%)
Religion				
Catholic	208	(36%)	123	(34%)
Muslim	148	(26%)	86	(24%)
Protestant	151	(26%)	86	(24%)
Born again	52	(9%)	58	(16%)
Other	12	(2%)	5	(1%)
Education				
None/Primary	163	(29%)	123	(34%)
Some secondary/O level	225	(39%)	162	(45%)
A level/vocational training/university	183	(32%)	73	(20%)
Able to read	546	(96%)	318	(89%)
Employed	530	(93%)	217	(61%)
Number of children				
None	199	(35%)	60	(17%)
1-2	207	(36%)	157	(44%)
3 or more	165	(29%)	141	(39%)
3 or more	165	(29%)	141	(39%)
Women’s past year physical IPV	–	–	32/354	(9%)
Women’s past year sexual IPV	–	–	58/354	(16%)
Relationship changed since exposed to SASA!	491/518	(95%)	213/354	(60%)
SASA! Exposure:				
Communication materials/poster				
Never*	3	(1%)	17	(5%)
1 time	94	(17%)	57	(16%)
A few times (2-4)	301	(53%)	85	(24%)
Many times (5+)	173	(30%)	199	(56%)
Drama/film (mid media)				
Never*	99	(17%)	121	(34%)
1 time	177	(31%)	75	(21%)
A few times (2-4)	198	(35%)	99	(28%)
Many times (5+)	97	(17%)	63	(18%)
Discussion activity (Interpersonal communication)				
Never*	60	(11%)	110	(31%)
1 time	179	(31%)	81	(23%)
A few times (2-4)	237	(42%)	114	(32%)
Many times (5+)	95	(17%)	53	(15%)
Sought CA advice (Interpersonal communication)				
Never*	354	(62%)	286	(80%)
1 time	125	(22%)	20	(6%)
A few times (2-4)	60	(11%)	36	(10%)
Many times (5+)	32	(6%)	16	(4%)
Multi-channel exposure vs. mass media only (*exposure to materials plus activities and/or films)*				
None*	1	(%)	2	(1%)
Mass media only	28	(5%)	80	(22%)
Low multi-channel exposure	283	(50%)	138	(39%)
High multi-channel exposure	259	(45%)	138	(39%)

*Given the sample, 'never' category here indicates participants with some SASA! exposure, but no exposure to the specified channel

### Communication channels and exposure to SASA!

In the quantitative sample, among partnered community members who reported at least some exposure to SASA!, nearly all had seen SASA! materials (e.g. posters) and 69% of women and 89% of men had been to a discussion activity at least once (Table 2). Drama exposure was also high (83% of men and 66% of women) and the majority had attended a few times at least. Nearly twice as many men (39%) report seeking advice from a community activist, compared to women (20%). And, most participants were exposed through multiple channels with 39% of women and 50% of men reporting low (1-4 times) ‘multi-channel’ exposure (materials plus drama and/or discussion activity exposure) and 45 and 39% (respectively) high exposure (5 or more times).

In the qualitative sample, 18 of the 20 participants had been exposed through at least one communication route, with two women reporting no exposure at all (5F, 8F). The intensity and type of exposure to SASA! varied among participants. There were examples of couples and individuals that primarily had direct relationship support from a community activist (2 M, 10F, 10 M), and others who only had attended activities or dramas (1F, 1 M, 5 M, 7F, 9 M, 9F). The former case tended to be couples who had been experiencing violence and either went to the local council office for support, or sought support from a friend, neighbour or relative that was a community activist. One woman did not feel motivated to attend activities because she received intensive support from the CA:

In that area [attending activities] I have been lazy, maybe it is because I was relying on [CA]...but still I cannot say that I am so informed about their activities...I get to hear about these things from [our CA]...he usually tells me that they have gone for training, things like that...but we have not been active in attending them. (10F).

**Fig. 3:**
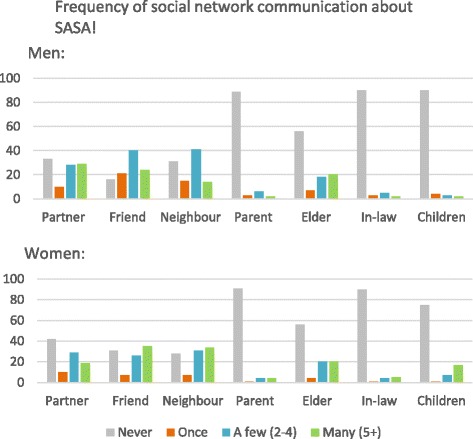
Frequency of social network communication about SASA!

The quantitative results strongly suggest SASA! is diffusing throughout intervention communities (Table 3 and Fig. 3), with between 69 and 85% of men and women in the sample reporting their friends, neighbours and elders attended SASA! activities. As for partners, 54% of men versus 14% of women report their partner attended, showing significant gendered variation. Large proportions of participants also report talking about SASA! with one or more members of their social network (83% of women and 92% of men) and the majority did so more than once. As for intimate partners, there was also a gendered variation as seen with attendance, with 67% of men versus 58% of women reporting speaking to their partner about SASA!. The results also indicate the diffusion of SASA! in the community beyond the sample: it is not only the participants initiating discussions about SASA!, but friends, neighbours and parents as well. Interestingly, elders are the only group that participants report initiated the conversation the majority of the time (72% of men and 68% of women reported this).

**Table 3:** Characteristics of social network participation and communication about SASA!

	Male		Female	
	n/N	(%)	n/N	(%)
Social Network Members Attending SASA! *(as reported by participants)*				
Partner	280/520	(54%)	50/348	(14%)
Friend	463/542	(85%)	248/342	(73%)
Neighbour	444/534	(83%)	282/334	(84%)
Parent	66/552	(12%)	31/342	(9%)
Elder	384/559	(69%)	245/332	(74%)
In-law	65/543	(12%)	30/335	(9%)
Children	171/550	(31%)	188/349	(54%)
Characteristics of communication with different social network members:				
Respondent talked to:	(*N* = 512)		(*N* = 289)	
Both sexes	382	(75%)	142	(49%)
Same sex only	122	(24%)	140	(48%)
Opposite sex only	8	(2%)	7	(2%)
Who initiated talks about SASA!:				
Network member initiated all talks	117/571	(21%)	96/358	(27%)
Partner initiated	96/376	(26%)	15/204	(7%)
Friend initiated	175/470	(37%)	82/246	(33%)
Neighbour initiated	187/389	(48%)	122/257	(48%)
Parent initiated	30/61	(49%)	14/32	(44%)
Elder initiated	180/249	(72%)	108/158	(68%)
In-law initiated	15/54	(28%)	11/37	(30%)
Children initiated	4/54	(7%)	38/89	(43%)

The qualitative data indicate interpersonal communication and materials were the main communication channels through which participants first became aware of and engaged with SASA!. Over half were first exposed when a community activist came to their home and invited them to join an activity or drama:

John [CA] mobilised us to come and attend…it even rained on that day but we went and attended...for us we just went because we were mobilised, we did not know what we were going to learn that day (4F).

The first time, there was a lady called Mukyala Mukulu (women representative on Local Council). And man called Musomesa are the ones who brought the SASA sensitizations/ activities in our community. We also joined and they taught us. (8 M).

As these quotes illustrate, being personally invited to join—especially by a known community member—was a strong motivating factor for many. For others, their first exposure came through informal discussions with members of their social network. This included casual discussions with other community members who had attended, seen posters or observed activities taking place in the community. And, as noted above, in a few cases couples’ first exposure came following an episode of IPV when the wife sought support at the local council or when a CA intervened. For example, one participant went to the Local Council (LC) to report her husband after a fight over his lack of financial support escalated to physical violence:

I went to the LC office and reported him and they called both of us...I wanted this man [her husband] to look after us, not that I reported him so that we could separate. (6F).

They were received and counselled by the Local Council leaders that were also trained CAs and with their encouragement the couple then went on to attend SASA! activities, dramas and video screenings over time.

Posters played an important role in raising general awareness of SASA! and promoting ongoing attendance. Participants often noted first seeing posters displayed around the community as they came to know about SASA!. The posters continued to have relevance over time as participants continued to review and reflect on the different relationship and family scenarios depicted (detailed later). The ‘loud speaker’ (community public announcement system) was influential in promoting ongoing attendance. Participants frequently spoke of continuing to attend whenever they heard activities announced on the ‘loud speaker,’ whereas their first exposure generally came from the other channels.

60% of women and 95% of men in the quantitative sample reported positive change in their relationship since becoming involved in SASA!. The data suggest interpersonal communication played the strongest role. Men were more likely to report positive changes in their relationship due to SASA! when they attended interactive discussion activities and spoke numerous times (5+) with their partner about SASA! (Table 4). There were also dose response relationships observed for both exposures, with the associations increasing between the low (1-4 times) and high frequency (5+ times) categories. For women, there were strong independent effects (and dose response relationships) for all exposures apart from talking with peers, seeking CA advice and drama attendance (*p*-value > .05 for these variables) (Table 4). Similar to men, frequent talks with their partner about SASA! had the strongest independent effect on relationship change (aOR 7.08, CI 2.29-21.90). However, women who talked with elders about SASA! were 9.62 times more likely to report relationship change, whereas for men there was no effect. Communication material exposure (aOR 4.3, CI 1.69-10.93) and discussion activities (aOR 3.53, CI 1.46-8.54) also showed strong independent effects on women reporting relationship change.

**Table 4:** Association between SASA! exposure and interpersonal communication and reporting positive change in relationship

	Female								Male							
Variable(Channel) (*N* = 354)	*n*	% reporting change	OR	95% C.I.	*p*-val*	aOR^a^	95% C.I.	*p*-val*	*n*	% reporting change	OR	95% C.I.	*p*-val*	aOR^a^	95% C.I.	*p*-val*
SASA! Exposure:																
Materials/posters					< 0.01			< 0.01					0.27			0.68
0-1 times	17/72	(24%)	1	–		1	–		72/79	(91%)	1	–		1.00	–	
Few times (2-4)	33/83	(40%)	2.14	(1.06-4.30)		1.15	(0.47-2.78)		267/272	(96%)	2.31	(0.86-6.16)		1.14	(0.36-3.63)	
Many times (5+)	163/199	(82%)	14.65	(7.63-28.14)		4.3	(1.69-10.93)		158/167	(95%)	1.71	(0.61-4.76)		0.68	(0.18-2.61)	
Drama/film (mid media)					< 0.01			0.49					< 0.01			0.41
Never	48/118	(41%)	1	–		1	–		69/79	(87%)	1	–		1.00	–	
Once	40/74	(54%)	1.72	(0.95-3.08)		0.52	(0.21-1.25)		144/155	(91%)	1.9	(0.77-4.68)		1.61	(0.56-4.68)	
A few (2-4 times)	68/99	(69%)	3.2	(1.82-5.61)		0.63	(0.26-1.55)		187/191	(98%)	6.78	(2.06-22.31)		3.45	(0.76-15.66)	
Many times (5+)	57/63	(91%)	13.85	(5.53-34.69)		0.8	(0.19-3.44)		91/93	(98%)	6.59	(1.40-31.07)		2.42	(0.37-15.76)	
Discussion activity (interpersonal comm)					< 0.01			0.02					< 0.01			0.03
Never	34/107	(32%)	1	–		1	–		35/43	(81%)	1	–		1.00	–	
1 time	47/80	(59%)	3.06	(1.67-5.59)		2.16	(0.90-5.18)		144/155	(93%)	2.99	(1.12-7.99)		3.47	(1.01-11.92)	
A few/many times (2+)	132/167	(79%)	8.1	(4.66-14.06)		3.53	(1.46-8.54)		312/320	(98%)	8.91	(3.15-25.23)		5.77	(1.52-21.95)	
Sought CA advice (interpersonal comm)					< 0.01			0.09					0.01			0.83
No	147/282	(52%)	1	–		1	–		282/304	(93%)	1	–		1.00	–	
Yes	66/72	(92%)	10.1	(4.24-24.06)		2.65	(0.84-8.34)		209/214	(98%)	3.26	(1.21-8.75)		1.13	(0.35-3.71)	
Multi-channel exposure	(*N* = 352)				< 0.01			< 0.01	(*N* = 518^1^)				< 0.01			< 0.01
Mass media only	24/78	(31%)	1.00	–		1.00	–		8/12	(67%)	1.0	–		1.00	–	
Low multi-channel exp. (1-4)	77/136	(57%)	2.94	(1.63-5.29)		3.26	(1.73-6.15)		238/255	(93%)	7.00	(1.91-25.61)		6.17	(1.49-25.47)	
High multi-channel exp. (5+)	112/138	(81%)	9.69	(5.10-18.43		12.3	(6.09-24.85)		245/251	(98%)	20.4	(4.80-86.86)		15.72	(3.22-76.74)	
Interpersonal Communication about SASA!:																
Talked to partner					< 0.01			< 0.01					< 0.01			0.02
never	46/147	(31%)	1	–		1	–		130/146	(89%)	1	–		1.00	–	
medium (1-4)	105/139	(76%)	6.78	(4.03-11.41)		3.05	(1.53-6.11)		202/212	(95%)	2.49	(1.09-5.65)		1.34	(0.47-3.84)	
high (5+)	62/68	(91%)	22.69	(9.15-56.23)		7.08	(2.29-21.90)		160/160	(99%)	19.6	(2.56-149.53)		13.10	(1.33-128.54)	
Talked to peers					< 0.01			0.35					0.01			0.26
low (0-2)	24/102	(24%)	1	–		1	–		127/142	(89%)	1	–		1.00	–	
medium (3-5)	32/50	(64%)	5.78	(2.77-12.07)		1.78	(0.71-4.46)		109/113	(97%)	3.22	(1.04-9.99)		2.49	(0.69-8.97)	
high (6+)	157/202	(78%)	11.34	(6.45-19.95)		1	(0.41-2.43)		255/263	(97%)	3.76	(1.56-9.11)		0.94	(0.30-3.01)	
Talked to elders					< 0.01			< 0.01					0.12			0.85
low (0-2)	71/190	(37%)	1	–		1	–		268/288	(93%)	1	–		1.00	–	
medium (3-5)	109/128	(85%)	9.62	(5.44-16.99)		4.05	(1.85-8.88)		185/185	(97%)	2.23	(0.88-5.65)		1.21	(0.37-3.89)	
high (6+)	33/36	(92%)	18.44	(5.45-62.32)		5.7	(0.99-32.71)		44/45	(98%)	3.28	(0.43-25.09)		0.60	(0.05-6.85)	

* Overall *p*-value estimation based on likelihood ratio test ^a^Controlled for age, marital status, education level, SES and other SASA! exposure

Overall for both women and men the data indicate exposure to *both* materials and activities or dramas/videos had the greatest effect. Men with low (1-4 times) and high (5 or more times) multi-channel exposure were respectively 6.17 and 15.72 times more likely to report relationship change following exposure to SASA! versus those with only materials exposure; and women were 3.26 and 12.3 times more likely to report this (Table 4).

### Intervention attributes and diffusion

A range of intervention attributes and change agent factors were key in promoting awareness of and engagement with SASA! activities and new ideas and, in some cases, behaviour change.

#### Observability

To start, there was a sense, even from participants with less exposure, that SASA! was a visible part of the fabric of the community. This ‘observability’ was evidenced by how participants noted “seeing people talking about it,” referenced those “who are active in it” and “put up posters” and described how “when you come back in the evening they [neighbours/family] will tell you that the SASA! team was here.”(1 M). There was a strong sense of collective engagement in SASA! illustrated in the way participants often discussed their participation as a community, rather than individual endeavour. For example, similar to this participant, when asked about their personal engagement with SASA! many responded using “we” language and spoke about it as a community endeavour: “All the women here attended...we went together”(1F). Both the awareness of SASA! activities and talk among community members stimulated curiosity and motivated attendance. For example, one participant described how his curiosity was piqued after hearing talk about SASA! in the community and led him to attend his first drama:

Interviewer: When you heard that there was a SASA! activity, what did you think about that?

Participant: “I had to know the meaning of SASA!, [people] would say that there are SASA! dramas there, and then I would ask myself that what is SASA!? Is it a drama? That prompted me …they had even told us that Nandujja [popular traditional dancer] was coming, she was the first one to come. I decided to go and watch her, when she finished then they brought a drama.” (5 M).

Here the participant demonstrates how SASA!‘s visible presence in the community—with most activities and dramas held in local gathering spaces—stimulated curiosity and encouraged engagement. After attending the drama he, like others, went on to attend different SASA! activities when offered. There was also the perception among many participants that SASA! was appreciated and well received by the wider community:

Many people, neighbours, those I work with like the Boda-Boda [motorcycle taxi] riders and even you can see that they like SASA! activities. (8 M).

(Though their narratives may have been exaggerated if they perceived the interviewer was associated with SASA!). This in turn led to a mutually reinforcing relationship, in that communication about SASA! motivated community members to attend and this generated more discussion. Social network members also played a role by encouraging each other to continue attending activities. As this participant notes, community members often helped spread the word about activities:

What encourages me to go there is the hope that they would bring a new idea…especially the ideas that help us on the things we are working on. And you will find colleagues who will tell you...‘SASA! sessions are going on.’ Because it has helped to create better families now. (3 M).

Within social networks informal conversations about and engagement with SASA! was important not only in motivating attendance, but in enhancing the observability of changes in couples who were involved in SASA!. This is indicated in the way participants frequently reported observing a reduction in IPV in their communities. They may have felt compelled to report changes in their community and, indeed, some accounts did appear superficial with vague, blanket statements that people had changed. However, others, like this participant, shared specific examples of couples they had seen change:

There is one that I saw who was not ‘seeing properly [not understanding],’ but when she participated in these SASA! activities….it helped to change their home/relationship, to know that violence is not their solution, I saw that. (5 M).

Seeing positive changes in neighbours and friends seemed to increase the perception of SASA! as an effective means of reducing IPV and improving family life:

Everyone you talk to, will always tell you that SASA! activities have changed life for the better. (8 M).

It was also emphasised how sharing and learning from each other’s experiences gave them a new perspective on how to handle challenges.

#### Compatibility and relative advantage

A central theme influencing exposure and engagement was SASA!‘s compatibility with participants’ lives and personal challenges. To begin, the close proximity of SASA! to participants’ communities and daily lives made it feel intimate and personal. Several remarked that SASA! was not like other programmes that “decide to stay at the health centres, where they sensitise the people from”(8 M). As this participant illustrates, there was a strong appreciation that SASA! activities came right to them:

They have even reached down to the grass root people, instead of people saying that they are going to watch a drama, the drama comes down to them. (5 M).

Second, participants found meaning through their identification and connection with the topics discussed at activities and observed in dramas and videos. They frequently noted activities reflected their own experiences and those around them. One participant explained:

[T]he information was good and I think it was like a lesson because we were also going through the same situation. (6F).

For many, seeing the cause and effect of scenarios that reflected their own lives also generated an affective (emotional) response and fostered new understanding. For example, one participant reported being most impacted by the stories depicted in the SASA! videos, explaining:

What has affected me most are the videos because they show you the beginning and the end, that if you do this, it shows you what the end result will be. (6 M).

Others described identifying with the content, to the degree they felt as though CAs “have come to talk about you specifically” and were moved in seeing their own behaviour – “every action that you do” – mirrored in dramas, videos and posters.

Third, the desire “to learn” was a primary motivation for activity attendance highlighted throughout the narratives. One participant noted she never received any education around being in a relationship and SASA! offered her an opportunity to learn. Here, another participant demonstrates the value many placed on learning and the potential relationship benefits that may follow:

I am a person who likes to learn new things. You know when you go for such activities you cannot be the same, even your marriage improves… it is like how we used to go to school, each day we would learn something new, I have learnt how to have a good relationship with my husband. (4F).

This also highlights the importance of participants’ ‘felt need’ for change in their relationship and whether they felt SASA! offered them enough advantages to attend and continue to engage. For example, one couple articulated how their attendance was directly linked to a desire for change in their volatile relationship:

I was motivated to come and attend that sensitisation activity about domestic violence...I wanted it to help me because the violence in my relationship was not ending...I thought that if the violence would reduce even in my home, even our relationship would become better. (6 M).

What motivated me is that they mobilised us and that they said that they were going to speak about violence and this is what was happening in my home…I had a problem in my home and I had to go and attend. (6F).

On the flip side some individuals who did not perceive that their relationship needed change because they had no physical IPV, were unmotivated to continue engaging with SASA! more actively—despite ongoing conflict, verbal abuse and/or controlling behaviour. For example, one participant exhibited controlling behavior, barred his wife from working and refused her requests to test for HIV, but noted SASA! was for those experiencing physical violence and not applicable to him:

Generally it would have been a good thing but...there are people like me, I personally never fight,... I personally don’t have problems in my relationship that would cause me to go there. Indeed if you had violence in your relationship you would. (1 M).

Other key barriers to engagement were a lack of proximity and incompatibility with participants’ lives. Some reported they did not attend because there were few activities in their area or the times were inconvenient:

I attended [only] one because they do not normally come to our community. (7F).

I wanted to attend their activities so that I listen to what they teach but I was not able to because I am busy working. But I thought that the next time when they come, I will attend and listen. (3F).

Suggestions were given that activities be held on Sunday or during times of the day when most people have finished work and household chores. However, these reasons may not be the full story, but socially acceptable responses. Participants may have wanted to portray a certain image to the interviewer to avoid, for example, showing a lack of interest in SASA!.

Lastly, while the loud speaker and door-to-door mobilisation were important communication channels in motivating attendance, some reported never hearing activities announced or that CAs failed to return following an initial visit. As these participants demonstrate, the lack of set times and advance notice of activities was a barrier to exposure for some and may impede diffusion in some cases:

[I]f you just come one morning and you walk through the community and tell people that come to the activity, you find that people already have their other programmes. (4 M).

It is difficult to tell somebody that you should go and participate in SASA! activities. That person will ask you ‘where are they?’ At that time it is difficult to answer that question...because we do not know... You just hear about it in the community that they [CAs] are coming, they [CAs] come and tell us that they are about to start... (5 M).

#### Change agent factors

Community activists played a central role in participants’ change process from their initial knowledge about SASA! ideas through implementation of new behaviours. Their role as change agents appeared to be particularly influential because they were part of community members’ social networks and also often respected ‘opinion leaders’. Several participants, mainly men, indicated pre-existing relationships with their community activists who were “resident[s],” friends, relatives or members of their local council. For example, one participant described how he had always “strongly admired” a CA, noting:

So because of that man being part of the SASA! team I wanted to listen and get to know whatever they were discussing. (1 M).

There was an appreciation that CAs were both part of the community – “one of us” – but also had links to outside networks as they “walk with the people from SASA!” and received training. Together this appeared to accord them value in the eyes of participants, legitimising their role and the new ideas they were sharing. This participant illustrates how CAs were able to reach people in casual and intimate ways because they were both respected members of their social network and often familiar with community members’ lives:

I saw him [CA] approaching me with a pile of materials. He gathered us together and said to me, ‘I am lucky I have met you because it is you who has married many women.’[teasing tone]...When we gathered he started asking us several questions. During the discussion I started telling him about family problems. In response he told me about the programme [SASA!] and that’s how I started knowing about those programmes. (10 M).

There were also cases that illustrated how a previous relationship with a CA or attributes of a CA can be a barrier to change. The most notable example was a participant who reported not being able to take his CA’s messages around SASA! seriously because of the nature of their long time friendship:

Joyce [CA] didn’t teach me...because we used to joke a lot and when sometimes she brought a topic [related to SASA!], I would think that she’s still joking so I failed to give her time that way. (9 M).

He also felt he could not go to her for support with his own relationship issues because of ongoing IPV in her relationship: “you cannot ask such a person for support because they are worse off” (9 M). This highlights a challenge surrounding the CA’s role in the community. On one hand observing their change/good relationship facilitated change in others, but on the other hand this can be a barrier when a CA is still experiencing violence in their own relationship, not modelling healthy relationship behaviours or has other attributes or beliefs that are not respected by community members.

And finally, many who engaged with SASA! and took on board the new ideas and behaviours (and some who did not) reported sharing what they had learned with others and, in some cases, becoming change agents in their own right. This was not dependent on extensive exposure or change, as diffusing SASA! messages was reported by participants with minimal exposure as well as those who had not applied much or any of the learning in their own life. Those with less exposure reported sharing ideas they had picked up from posters or seeing an activity once, or referring people to SASA! after hearing about it from others. Those with more exposure exhibited a deeper motivation to tell others about SASA! so they could also experience the benefits they enjoyed. For example, one participant, who experienced profound changes in his relationship due to SASA!, reported actively passing his learning on to others in the community:

“[SASA!] has helped to create better families now...we have actively participated to the extent that if you get like five people, we help them...so that they also learn and train others and this has increased the number of peaceful homes here.” (3 M).

The external visibility of change in couples also appeared to make them an attraction, with some more active participants assuming a role similar to a CA within the community (and beyond in some cases). This new status was meaningful for them and reinforced their own changes and desire to continue engaging with SASA! and sharing their learning to help others.

## Discussion

The findings indicate materials and mid media channels generated awareness and knowledge, while the concurrent influence from interpersonal communication with CAs and different social network members more frequently facilitated changes in behaviour or ‘adoption’. The most influential attributes of the intervention were its observability, compatibility and the relative advantage it provided to community members. Broadly, exposure through multiple communication channels was most effective in facilitating change. Those with low and high ‘multi-channel’ SASA! exposure had much higher odds of reporting relationship change than those with exposure to mass media materials only. In addition, the dose-response relationship observed suggests those with more ‘multi-channel’ intervention exposure were more likely to experience relationship change. While it is difficult to ground the findings in the context of other IPV studies given the dearth of research examining diffusion, ‘what works’ reviews of IPV intervention evidence have also found programmes which combine mass media messaging and community mobilisation with more interpersonal engagement (i.e. interactive group activities and individual counselling) are more effective in generating behaviour change [11, 40].

Dramas and videos (mid media channels) appeared to generate identification among participants and understanding of the causes and effects of IPV. This is evidenced by the narratives on realistic storylines facilitating identification while also modelling alternative perspectives and behaviours. This underscores the importance of developing program content that closely reflects the lives of people in the specific context. The concurrent influences of interpersonal communication with CAs and community members then appeared to give the media messages credibility, facilitating favourable attitudes towards them and encouraging behaviour change. Consistent with many diffusion studies [20, 25, 41], this concurrent influence was the most influential factor in the uptake of new ideas and behaviours. Our quantitative data showed interpersonal communication channel exposures alone (i.e. discussion activities with a CA, seeking CA advice, and talk about SASA! with partner, peers and elders) were associated with relationship change after controlling for the effect of all exposures, whereas materials and mid media exposure were not.

The findings also suggest talk about SASA! among peers may raise awareness and motivate attendance, whereas discussions with elders and partners may be more influential in changing their behaviour in their relationships. Talking with elders (among women) and one’s partner about SASA! showed independent effects with relationship change, whereas talking with peers did not. Diffusion research indicates those who are more similar to an individual are more influential in persuading them to adopt new ideas and behaviours [20]. Thus, it is interesting that talk with peers about SASA! did not have an independent effect, while talk with elders, who are in theory less similar, did. This may be due to the respected role elders have in Ugandan society regarding relationship guidance. This finding may also be attributed to ‘ssengas’ or paternal aunts who traditionally provide Baganda women guidance on relationships [42] as some were sensitised as part of the intervention. While there is currently a growing call within IPV prevention to focus efforts on younger populations [43], these findings support the simultaneous engagement of older generations, at least in culturally similar settings. During formative research and intervention design or adaptation it may be helpful for practitioners to, 1) examine who advises on and influences relationships in the given context (e.g. peers, elders, religious leaders, local leaders, schools) and ensure the intervention engages them as well as youth, 2) consider how to deliberately promote talking between partners about their relationship and what they are learning.

The intervention’s engagement of community members as change agents (i.e. community activists) appeared particularly impactful, because the ideas were emerging from trusted and known friends and neighbours rather than people from outside the community. The qualitative data indicates that CAs’ influence stemmed from the multiple roles they embodied within the community’s social network as community member (“one of us”), opinion leader and change agent. In our broader qualitative evaluation [22] we found CAs viewed their role as a vocation irrespective of formal programme support. It was a means through which they gained a valued identity, particularly for those who had not completed their formal education or achieved anything of note in their community. These findings are in agreement with diffusion theory and studies that found identifying opinion leaders and using them as change agents can increase diffusion of health promotion interventions at the community level [20, 41, 44]. However, similar to our findings, others have found the identification of actual opinion leaders was essential to their effectiveness [45]. Valente and Pumpuang studied methods for identifying opinion leaders and found programs using multiple methods such as expert identification and peer nominations were most likely to secure successful opinion leaders for promoting behaviour change [20, 41, 44].

Overall, our findings point to the value of using diffusion of innovations theory in the evaluation of IPV interventions. Intervention research often examines the effect of exposure to different aspects of the intervention on the intended outcomes, but stops there. Our findings demonstrate the vital role played by CAs as well as the importance of the intervention’s attributes of observability, compatibility and relative advantage—whereas relative findings for trialability and complexity did not emerge in the qualitative data. These insights made an important contribution to our understanding of *how* the intervention worked to influence behaviour change and may have been missed if the study was guided by an individual behaviour change model alone (e.g., transtheoretical model [46]) as they do not account for community level intervention and social network factors. The study on the Stepping Stones noted earlier illuminates this point: through examining diffusion the study found the messages were not spreading to the wider community, illuminating an important limitation that can be used to inform future interventions [19]. As Kippax and Stephenson contends we must design research to elucidate the ways individuals engage with interventions/messages and capture the mechanisms of change in order to find out what worked to improve interventions [47]. Diffusion of innovations theory should be given strong consideration both when developing and researching community mobilisation interventions like SASA! which are designed to diffuse through community social networks and change agents.

### Limitations

The study had a number of limitations. First, using data collected post-intervention introduces the potential for increased social desirability bias, especially when relying on self-reported attitudes and behaviours specifically promoted by the intervention. Participants may have exaggerated the impact of SASA!, out of a desire to please the investigators and present the programme in a positive light. While the qualitative data were interrogated across cases for the level of detail provided verses general statements of change—and triangulated with partner account where possible—we cannot exclude the potential influence of social desirability bias in their responses. Second, given the dynamic context, other factors may also influence changes in relationships such as: ssengas not sensitized by SASA! [42, 48] and messaging from HIV prevention campaigns, religious groups and other anti-violence initiatives in Kampala on trust, love and morality [48, 49].

Third, the single interview design was a limitation. Collecting data at multiple time points through longitudinal or pre/post interviews both places less reliance on recall and allows the researcher to observe across interviews the consistency in participant’s accounts of changes experienced, increasing validity. Participant’s ability to recall the ways they engaged with the intervention—sometimes over several years—likely impacted the data. While a longitudinal design would have been preferable, it was not deemed feasible due to the mobile characteristics of the study communities, resource constraints and other factors related to the RCT design. The qualitative study helped offset the recall challenge some as it was designed to capture the sequence of events and relationship changes over time using the participatory timeline tool. And, fourth, to avoid contamination in the RCT mass media (e.g. radio, TV) was not used and our conceptual framework had to be altered to reflect this. Poster exposure proved difficult to classify in the quantitative analysis as they were both displayed in the community and used during some discussion activities. This made it less clear cut what was communication materials/media exposure versus interpersonal communication exposure. While this required the quantitative results to be interpreted with caution, the qualitative data helped to tease this out more.

## Conclusions

This study contributes to our knowledge of IPV prevention in a low-income, urban East African context. Specifically, it highlights how using a community mobilisation approach that includes content that reflects peoples’ lives and direct leadership of local change agents can facilitate diffusion and powerful collective change processes in communities. Overall, this study makes clear the value of applying diffusion of innovations theory using mixed methods to illuminate the *how* as part of complex public health intervention evaluations.

## Abbreviations

aOR: Adjusted odds ratio
CA: Community activist (in the SASA! intervention)
CEDOVIP: Center for Domestic Violence Prevention
CI: Confidence interval
IPV: Intimate partner violence
LC: Local council
LRT: Likelihood ratio test
RCT: Randomised control trial
SES: Socioeconomic status
VAW: Violence against women
